# Detection of Benzo[*a*]pyrene Diol Epoxide Adducts to Histidine and Lysine in Serum Albumin In Vivo by High-Resolution-Tandem Mass Spectrometry

**DOI:** 10.3390/toxics10010027

**Published:** 2022-01-08

**Authors:** Javier Zurita, Hitesh V. Motwani, Leopold L. Ilag, Vassilis L. Souliotis, Soterios A. Kyrtopoulos, Ulrika Nilsson, Margareta Törnqvist

**Affiliations:** 1 Department of Environmental Science, Stockholm University, SE-106 91 Stockholm, Sweden; javier.zurita@ki.se; 2 Department of Materials and Environmental Chemistry, Stockholm University, SE-106 91 Stockholm, Sweden; leopold.ilag@mmk.su.se (L.L.I.); ulrika.nilsson@mmk.su.se (U.N.); 3 Institute of Chemical Biology, National Hellenic Research Foundation, 11635 Athens, Greece; vls@eie.gr (V.L.S.); skyrt@eie.gr (S.A.K.)

**Keywords:** polycyclic aromatic hydrocarbons, metabolism, liquid chromatography-mass spectrometry, protein adducts, human exposure

## Abstract

Electrophilic diol epoxide metabolites are involved in the carcinogenicity of benzo[*a*]pyrene, one of the widely studied polycyclic aromatic hydrocarbons (PAHs). The exposure of humans to this PAH can be assessed by measuring stable blood protein adducts, such as to histidine and lysine in serum albumin, from their reactive metabolites. In this respect, measurement of the adducts originating from the genotoxic (+)-anti-benzo[*a*]pyrene diol epoxide is of interest. However, these are difficult to measure at such low levels as are expected in humans generally exposed to benzo[*a*]pyrene from air pollution and the diet. The analytical methods detecting PAH-biomarkers still suffer from low selectivity and/or detectability to enable generation of data for calculation of in vivo doses of specific stereoisomers, for evaluation of risk factors and assessing risk from exposures to PAH. Here, we suggest an analytical methodology based on high-pressure liquid chromatography (HPLC) coupled to high-resolution tandem mass spectrometry (MS) to lower the detection limits as well as to increase the selectivity with improvements in both chromatographic separation and mass determination. Method development was performed using serum albumin alkylated in vitro by benzo[*a*]pyrene diol epoxide isomers. The (+)-anti-benzo[*a*]pyrene diol epoxide adducts could be chromatographically resolved by using an HPLC column with a pentafluorophenyl stationary phase. Interferences were further diminished by the high mass accuracy and resolving power of Orbitrap MS. The achieved method detection limit for the (+)-anti-benzo[*a*]pyrene diol epoxide adduct to histidine was approximately 4 amol/mg serum albumin. This adduct as well as the adducts to histidine from (−)-anti- and (+/−)-syn-benzo[*a*]pyrene diol epoxide were quantified in the samples from benzo[*a*]pyrene-exposed mice. Corresponding adducts to lysine were also quantified. In human serum albumin, the anti-benzo[*a*]pyrene diol epoxide adducts to histidine were detected in only two out of twelve samples and at a level of approximately 0.1 fmol/mg.

## 1. Introduction

Polycyclic aromatic hydrocarbons (PAHs) are widely present in the environment as a result of incomplete combustion of organic matter. Common exposure sources for the general population of PAHs are cigarette smoke, vehicle exhausts and residential wood burning [1,2], as well as intake of grilled and smoked food [3,4]. Several PAHs are animal carcinogens and classified as possibly or probably carcinogenic to humans. One of the most well studied PAHs is benzo[*a*]pyrene, a PAH consisting of five fused benzene rings and classified as a human carcinogen [5].

Following exposure, PAHs can be metabolized by cytochrome P450 and epoxide hydrolase to the corresponding diol epoxides. These electrophilic metabolites have a potential to cause mutations depending on their ability to react with DNA [2,5,6] and are able to react with nucleophilic sites of amino acids in blood proteins. Benzo[*a*]pyrene diol epoxides (BPDEs) are relatively well-studied in this respect. In pioneering studies, the research group of Tannenbaum et al. showed that adducts from BPDE are formed to serum albumin (SA) of humans [7,8] by reaction with the carboxylate side chains of glutamic and aspartic acid and the imidazole ring of histidine (His) 146, as well as with the carboxylate groups in hemoglobin (Hb) [9].

Analytical methods based on mass spectrometry (MS) have previously been developed and applied to measure specific human exposures to short-lived electrophilic compounds/metabolites in vivo by measuring their adducts to the abundant blood proteins, SA or Hb [10,11,12]. Use of the most abundant blood proteins, instead of DNA, to measure adducts as biomarkers of exposure and to assess the internal dose is an advantageous strategy considering that the proteins have long half-life in humans and that the adducts are not repaired. This means that chemically stable adducts are accumulated during chronic exposure to the chemicals, depending on elimination kinetics of the respective protein [10].

Over the years, attempts to identify and measure adducts from BPDE to human SA and Hb as biomarkers for in vivo doses from benzo[*a*]pyrene exposure have been carried out using various analytical techniques (reviewed by Käfferlein et al.) [13]. Methods based on gas chromatography (GC)/MS have been used to measure tetrols originating from hydrolysis of BPDE adducts in human SA or Hb. Limitations of these methods include the fact that the carboxylic group adducts from BPDE are chemically unstable both in vivo and in vitro [14] and that there is no specificity of the origin of the analyte, which together make accurate quantification of the adduct level in relation to exposure uncertain. Previous studies of BPDE-adducts with laser-induced fluorescence [15] or GC/MS [13] have reported adduct levels ranging from less than 50 amol to a few fmol per mg of human SA. In spite of this, the existing methodologies were considered unsatisfactory in both selectivity and required detection limits (LODs) and were often not able to differentiate between exposed and non-exposed populations [13,16]. Among the methods is the enzyme-linked immunosorbent assay (ELISA), utilizing the BPDE antibody 8E11. Later, a sandwich ELISA method using the same antibody was shown to improve the measurement of intact SA adducts in human plasma and quantification at fmol/mg level was accomplished [17]. However, a drawback with ELISA methods for quantification is the risk of antibody cross-reactivity, i.e., other PAH metabolites could be interfering [13], thus contributing to measured adduct levels.

We have previously measured stable adducts from BPDE isomers in human SA in vitro, where cleavage of the protein was performed using hydrazine [18] or enzymatic digestion [19,20], followed by high-performance liquid chromatography (HPLC)-MS analysis. The major site of adduct formation in SA was found to be His146, while adducts to lysine (Lys) 195 were detected to a minor extent. These binding sites have been confirmed by proteomics [17]. In the early work by Tannenbaum et al., it was shown that (−)-anti enantiomer of BPDE binds to N^τ^ in His in SA [7] and that (+) and (−) enantiomers of anti-BPDE exhibit different reactivity towards the nucleophilic sites in SA [8,13]. We have shown in vitro that (+)-anti-BPDE as well as (+/−)-syn-BPDE form adducts to His in SA [20,21]. It has earlier been shown that the (+)-anti enantiomer of BPDE is the most tumorigenic metabolite [13,22]. Therefore, it is desirable that the analytical method is able to distinguish adducts from this specific metabolite from the other BPDE enantiomers. In our earlier work, we have been able to detect BPDE adducts to His in SA from mice exposed to high doses (100 mg/kg body weight) of benzo[*a*]pyrene [21,23]. Adducts to His were monitored with HPLC/electrospray ionization (ESI)-MS/MS, using triple quadrupole MS, as BPDE-His-Pro after pronase digestion. However, regarding the detection in human samples in vivo, the results from earlier studies show that at least two orders of magnitude lower LODs would be required [21,23]. A higher chromatographic selectivity to reduce the matrix interferences and to improve the separation of the adduct isomers would be useful to lower the detection limits.

**Figure 1:**
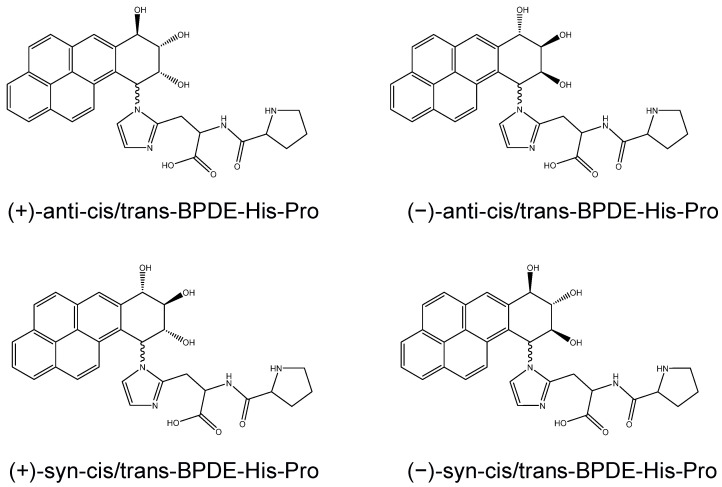
Structures of the studied His adducts in SA from (+/−)-anti-BPDE and (+/−)-syn-BPDE.

HPLC/ESI-MS/MS methodologies have significantly broadened the range of applications related to modified amino acids in general. The possibility to determine and characterize amino acid adducts to trace exposure to reactive electrophilic chemicals across the general population, such as from dietary intake or workplace environments, has paved the way for adductomics [24,25,26,27]. A highly selective MS platform that combines high-resolution, high *m*/*z* accuracy and ion fragmentation (tandem HRMS) would increase the possibility to identify and measure such trace levels of adducts, as has been reported in studies with DNA adducts [28,29,30]. Here, we present a methodology based on HRMS, including improved HPLC separation, to detect and identify amol–fmol/mg level adducts from BPDE to SA in vivo, after protein digestion and solid-phase extraction (SPE) clean-up. To ensure identification of the isomers, spiked samples were eluted on two different HPLC columns of different retention characteristics. Of particular interest was to study adducts to both His and Lys from (+)-anti-BPDE, that is, anti-7R,8S-dihydroxy-9S,10R-epoxy-7,8,9,10-tetrahydrobenzo[*a*]pyrene (cf. Figure 1). SA alkylated in vitro by BPDEs was used for method development and evaluation. The method was tested for its applicability to SA samples from mice exposed to benzo[*a*]pyrene and from humans without known occupational exposure.

## 2. Materials and Methods

### 2.1. Chemicals

Protease from *Streptomyces griseus* (pronase) and benzo[a]pyrene were obtained from Sigma Aldrich (St. Louis, MO, USA). (±)-Anti-, (±)-syn- and (+)-anti-benzo[a]pyrene-7,8-diol-9,10-epoxide (BPDE) were purchased from the National Cancer Institute (NCI), Chemical Carcinogen References Standard Repository (Kansas City, KS, USA). Ammonium bicarbonate of 99% purity was purchased from VWR International (Radnor, PA, USA). All solvents were suitable for HPLC and purchased from VWR International. All water used was of ultrapure quality (resistivity 18.2 MΩ cm) and obtained with a Milli-Q purification system (Merck Millipore, Molsheim, France). Eppendorf Protein LoBind tubes (Hamburg, Germany) were used.

### 2.2. In Vivo Serum Albumin Samples from Mice and Humans

An experiment to obtain biological samples from benzo[*a*]pyrene-treated mice was performed at the National Hellenic Research Foundation, Athens, after approval from their ethical committee [21]. The biological material for the present study was selected from a larger animal study. For the present study, male mice (wild-type CD2F1, 25–30 g, 10 weeks old, obtained from Charles River Laboratories Italia S.P.A., Italy) treated with the highest dose of benzo[*a*]pyrene (100 mg/kg body weight, dissolved in tricaprylin, administered with single intraperitoneal injection) were selected. The mice were sacrificed after ether anaesthetization at 1, 3, 7, 14 and 28 days after treatment for collection of samples for studies of benzo[*a*]pyrene metabolism and adduct formation in mice. Samples from all time points were used to be able to follow formation and disappearance of adducts. Blood samples were collected in heparinized tubes; the samples were centrifuged to isolate plasma, which was then stored at −20 °C and shipped to Sweden for analysis. In the present study, samples from two mice from each day of sacrifice were used for analysis for BPDE-adducts. SA from unexposed mice was used as control.

SA was isolated from plasma with saturated ammonium sulphate as earlier described [21]. Briefly, the whole blood samples was centrifuged (3000× *g*, 10 min), and the supernatant containing plasma (80−150 μL in the individual samples) was separated from the pellet of red blood cells. SA was isolated from plasma by slow addition of saturated ammonium sulphate (1:1 *v*/*v*, 6 °C) followed by centrifugation (12,000× *g*, 10 min, 4 °C) to precipitate the globulins. SA in the supernatant was precipitated with 0.5 M acetic acid (pH 4) and centrifuged (12,000× *g*, 10 min, 4 °C). The precipitate containing the SA was washed stepwise with methanol, ethyl acetate and pentane, followed by centrifugations (3000× *g*, 10 min, 4 °C) after each step. The precipitate was left to dry overnight at room temperature. In the earlier study the content of SA in the precipitate (which includes salt) was measured to 46–60% in the samples, by spectrophotometer using UV absorbance at 280 nm^21^. Ten-milligram samples of the precipitated mouse SA were digested and subjected to SPE as described below (Section 2.3) for the in vitro BPDE-alkylated human SA, and finally analyzed for BPDE-His-Pro and BPDE-Lys isomers by HPLC/Orbitrap MS/MS (cf. Section 2.4).

Twelve human blood samples were collected with ethical approval from the Regional Ethical Review Board in Stockholm (nr 96–312). Briefly, plasma was separated by centrifugation (4500× *g*, 10 min) and stored in the freezer (−20 °C) until SA was precipitated, as described above. Ten-milligram samples of the precipitated SA from humans were treated similarly as described for the mice samples and were analyzed by HPLC/ESI-Orbitrap MS/MS.

In the present work, we study BPDE adducts to SA from both mouse and human. From our previous studies, it is indicated that His146 in SA in the two species have similar reactivity. In vitro alkylation with (±)-anti BPDE gives approximately the same adduct levels to His in mouse SA and in human SA, as well as the same ratio (see Section 2.3) of adduct levels from (+)- and (−)-anti-BPDE [20,21]. Further, the peptide sequence in SA close to His146 is identical in the two species, which indicates there is no large difference in the adduct forming capacity of His 146 [21].

### 2.3. Preparation of a Stock Solution of Reference Adducts from In Vitro BPDE-Alkylated Serum Albumin

Samples of human SA had previously been alkylated in vitro with (±)-anti-BPDE and (±)-syn-BPDE, respectively [20]. The general procedure was that BPDE solution in tetrahydrofuran was mixed with human SA (80 mg) in water (2 mL) to give a final solution of 0.5 μg BPDE per mg SA. The pH was adjusted to 7.5 with NaOH (0.1 M), and the mixture was incubated at 37 °C for 24 h. Subsequently, the mixture was adjusted to pH 4 with 1 M HCl, and saturated ammonium sulphate (2 mL, 4 °C) was added to precipitate the SA. The precipitate was isolated, washed with methanol, ethyl acetate and pentane and dried at room temperature. The adduct levels determined in the earlier work [20] by HPLC/UV of (+/−)-anti-BPDE-His, (+/−)-anti-BPDE-Lys, (+/−)-syn-BPDE-His and (+/−)-syn-BPDE-Lys were 1.4, 1.4, 1.3 and 1.9 pmol/mg human SA, respectively. In the present work, these alkylated SA samples were used after digestion to obtain reference compounds (cf. Figure 1). For the digestion, 1 mg of each alkylated SA was digested with 500 µL of pronase (2 mg/mL) in ammonium bicarbonate buffer (50 mM, pH 7.0) at 37 °C for 20 h.

Following the enzymatic hydrolysis, generated BPDE-His-Pro and BPDE-Lys were enriched using Sep-Pak Plus C_18_ cartridges (Waters Corporation, Milford, MA, USA) from the different protein digests, according to the method described by Westberg et al. [19]. SPE clean-up was performed for each of the two alkylated SA digests (with (±)-anti-BPDE and (±)-syn-BPDE, respectively). Briefly, the SPE protocol was as follows: conditioning with 5 mL each of methanol (MeOH) and water, before loading the human SA enzymatic digest; washing with 5 mL each of sodium acetate (0.5 M, pH 5.0), water and 30% MeOH (aq); elution with 2 mL of 95% MeOH (aq). Each extract was dried and reconstituted in 350 µL of water. The two resulting separate solutions were then mixed to give one common stock solution with 4.0 fmol/µL of (+/−)-anti-BPDE-His-Pro, 4.0 fmol/µL of (+/−)-anti-BPDE-Lys, 3.7 fmol/µL of (+/−)-syn-BPDE-His-Pro, and 5.4 fmol/µL of (+/−)-syn-BPDE-Lys. The combined stock solution was diluted to obtain the working solutions for the calibration curves at 6 different concentrations. From our previous work [20] with these adduct standards, it is known that the ratio of His-adducts from (+)-anti-BPDE to those from (−)-anti-BPDE is 1:9 and that of Lys-adducts 3.5:1. The concentration of (+)-anti-BPDE-His-Pro and (−)-anti-BPDE-His-Pro in the prepared stock solution was thus estimated to be 0.4 and 3.6 fmol/µL, respectively, and that of (+)-anti-BPDE-Lys and (−)-anti-BPDE-Lys was estimated to be 3.1 and 0.9 fmol/µL, respectively. The concentration of individual (+)-syn- and (−)-syn-BPDE-adducts (to His and Lys) could not be determined due to lack of corresponding standards, and hence, we used the total levels of the (+/−)-syn adducts.

### 2.4. HPLC/ESI MS/MS of BPDE Adducts

The instrument used for identification of SA adducts from (+/−)-anti-BPDE and (+/−)-syn-BPDE was a QExactive™ Plus Hybrid Quadrupole-Orbitrap™ (Thermo Fisher Scientific, Waltham, MA, USA) equipped with an UHPLC UltiMate™ 3000 Standard Quaternary system (Thermo Fisher Scientific, Waltham, MA, USA). HPLC separation was performed on two different chromatographic columns, an Acquity UPLC BEH column C_18_ (50 × 2.1 mm, 1.7 µm, Waters Corporation, Milford, MA, USA) and a Kinetex F5 column (pentafluorophenyl, 100 × 2.1 mm, 2.6 µm, Phenomenex, Torrance, CA, USA). The LC condition applied in our earlier work [20,21] was used with some modifications. Briefly, a binary solvent system was used for both columns: solvent A was water/MeOH (95:5, *v*/*v*), and solvent B was water/MeOH (5:95, *v*/*v*). Formic acid was added to both solvents A and B at a concentration of 0.1%. The mobile phase composition for both columns was 10% B during the initial 0.6 min when the flow was diverted to the waste. This step was followed by a linear gradient from 10 to 100% B in 18.9 min and then 100% B for 5 min. Before next run, the column was re-equilibrated for 4.5 min under initial conditions. The flow rate was 0.4 mL/min and the injection volume 20 µL. 

MS analyses were performed in positive ESI mode with acquisition in parallel-reaction monitoring (PRM) mode. Protonated BPDE-adducts (*m*/*z* 449.2 and 555.2 for BPDE-Lys and BPDE-His-Pro, respectively) were chosen as precursor ions for MS/MS. The precursor isolation window was set to 1 *m*/*z*. The main fragment formed from both precursors was the same, measured at *m*/*z* 257.0961. Reference solutions, generated from digested BPDE-alkylated human SA as described in Section 2.3, were used for optimization of the MS parameters. The conditions set for the ion source were as follows: spray voltage 4.50 kV, capillary temperature 300 °C, auxiliary gas temperature 500 °C, sheath gas and auxiliary gas flow rate 20 and 10 arbitrary units, respectively, sweep gas flow rate 0 arbitrary units. S-lens RF level was set to 60; resolution (R = m/Δm) was set to 120 000 (FWHM) and automatic gain control (AGC) to 5 × 10^4^ counts. Nitrogen was used as collision gas and collision energy (high-energy collisional dissociation, HCD) was set to 45 eV. Thermo Xcalibur software v 3.1 was used for data acquisition set up and data processing as well as to obtain information on the elemental composition of the analytes.

The performance of the Orbitrap HRMS was compared with a triple-quadrupole instrument (Xevo TQ-S Micro from Waters Corporation, Milford, MA, USA) in terms of detectability of (+/−)-anti-BPDE-His-Pro and (+/−)-anti-BPDE-Lys. Conditions set for the ion source were as follows: capillary voltage 3 kV, source temperature 150 °C, desolvation temperature 300 °C, cone voltage 25 V, cone gas flow 50 L/h, and desolvation gas flow 800 L/h. Dwell time was set to automatic. Multiple reaction monitoring (MRM) transitions (with respective CE in brackets) monitored were 555.0 > 253.3 (30 eV), 555.0 > 257.3 (45 eV), 555.0 > 285.3 (40 eV) and 555.0 > 303.3 (30 eV) for BPDE-His-Pro adducts and 449.4 > 257.3 (30 eV) and 449.4 > 303.3 (30 eV) for BPDE-Lys adducts. Isolation width was set to 0.1 Da, full width at half maximum (FWHM). Nitrogen was used as collision gas, and collision gas flow was set to 0.15 mL/min.

### 2.5. Method Evaluation Using Reference Compounds

The combined stock solution with reference adducts from both (±)-anti-BPDE and (±)-syn-BPDE, obtained as described in Section 2.3, was diluted stepwise to provide working standard solutions. Linearity and instrumental limit of detection (LOD) were evaluated for each measured adduct isomer by preparing calibration curves at 6 different levels (single point) ranging from 0.1 to 10 fmol injected on the F5 column. The method LODs were obtained from the instrumental LODs estimated at S/N = 3, with N defined as the standard deviation from five replicates of a diluted working solution containing 14 fmol/mL of (+/−)-anti-BPDE-His-Pro, 68 fmol/mL of (+/−)-anti-BPDE-Lys, 13 fmol/mL of (+/−)-syn-BPDE-His-Pro, and 92 fmol/mL of (+/−)-syn-BPDE-Lys. Linearity was assessed by obtaining individual R^2^ from the calibration curves, and mass accuracy was assessed by measuring the difference between the observed *m*/*z* values and theoretical *m*/*z* values.

## 3. Results and Discussion

### 3.1. Orbitrap High-Resolution Mass Spectrometry

The BPDE adducts to His and Lys in SA were monitored with Orbitrap MS at instrumental settings described in Materials and Methods, Section 2.4. The use of pronase for the enzymatic digestion resulted in the His adducts being measured as BPDE-His-Pro (cf. Westberg et al. [19]). To evaluate the mass accuracy of the analysis, corresponding standard adducts from the in vitro (±)-anti-BPDE-alkylated human SA were used. The *m*/*z* values for each of the adduct isomers obtained with HRMS are given in Table 1. As shown, the deviations from theoretical *m*/*z* (Δ ppm) were below 1 ppm for all measured analytes. The accurate *m*/*z* values agreed with the elemental composition of the targeted ions, as well as with the double bond equivalents (DBEs).

**Figure 2:**
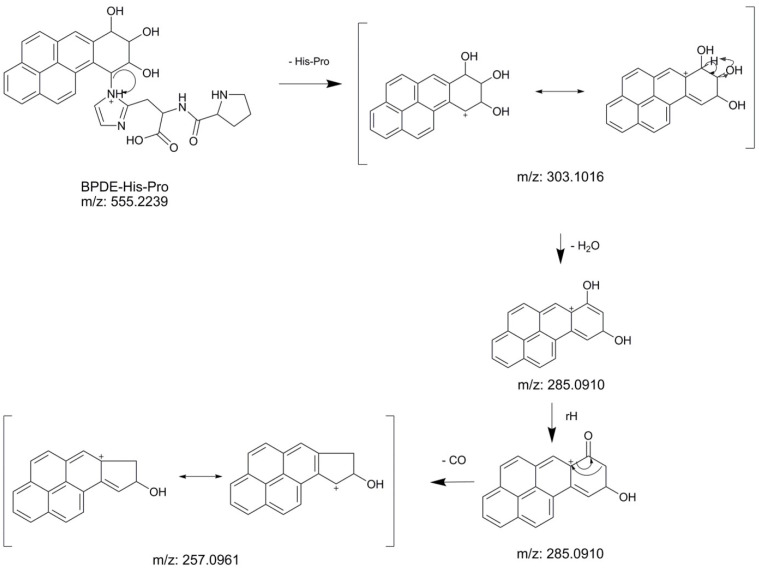
Fragmentation mechanism proposal for BPDE-His-Pro with theoretical *m*/*z* values given.

For higher selectivity and further verification of the identities, MS/MS was used to monitor the common protonated fragment at *m*/*z* 257.0961, formed after loss of His-Pro/Lys moiety, water and carbon monoxide. In Figure 2, a proposed mechanism for the fragmentation of BPDE-His-Pro is shown. The MS/MS spectra showing the fragmentation of BPDE-His-Pro and BPDE-Lys are provided in the Supplementary Material (Figure S1). Because of the combination of high resolving power, mass accuracy and specific MS/MS ion transitions, possible interferences were found to be almost eliminated from the chromatogram (illustrated under Section 3.2).

As described in Materials and Methods, Section 2.4, a low-resolving triple-quadrupole MS was also tested for comparison purposes. Significantly higher noise levels were obtained compared with the Orbitrap instrumentation. A comparison, using the same chromatographic column (C_18_), is shown in the Supplementary Material, Figures S2 and S3. While the triple-quadrupole exhibits about unit resolution over the entire *m*/*z* range, the Orbitrap has a clear advantage as it is able to distinguish targeted adducts from interfering compounds differing only by 1 ppm in molecular weight.

### 3.2. Chromatographic Separation of Adducts

SPE with C_18_ was applied in this work, mainly as a clean-up from non-modified amino acids. No further optimization was performed for the step of sample enrichment by SPE, as it was judged to be good enough based on earlier work [19]. The separation on C_18_ is based primarily on dispersion forces between the large hydrophobic BPDE moiety in the analytes and carbon chains of the stationary phase. This SPE method is therefore not to be regarded as highly selective and some of the non-modified amino acids having hydrophobic side chains might have been retained as well.

**Figure 3:**
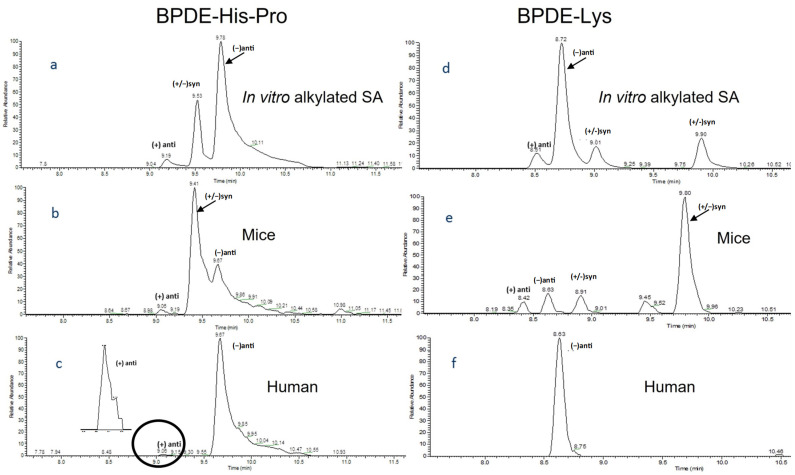
Extracted ion chromatograms showing separation of the studied adducts on an F5 HPLC column and using Orbitrap tandem HRMS (PRM mode). (**a**) BPDE-His-Pro from in vitro alkylated SA (1 mg); (**b**) BPDE-His-Pro from 10 mg SA of benzo[*a*]pyrene-treated mice (100 mg/kg of body weight) 1 day after exposure; (**c**) BPDE-His-Pro in human SA (10 mg) in vivo; in (**d**–**f**) are shown corresponding chromatograms for BPDE-Lys. The inset of Figure 3c shows a ×40 magnification of the peak corresponding to (+)-anti-BPDE-His-Pro (for retention times, see also Table 2). circled region shows the peak corresponding to (+)-anti-BPDE-His-Pro.

In the following HPLC analysis, usage of a pentafluorophenyl (F5) column was shown to significantly increase the chromatographic separation compared to a C_18_ column, which is important in order to avoid misidentifications but also for reduction of matrix effects on the ionization. The retention behavior of F5 is based on a combination of mechanisms, such as the interaction between the electron-deficient aromatic structure of the stationary phase and the electron-donating BPDE moiety in the analytes [31,32]. Figure 3a,d show the separation of the stereoisomeric BPDE adducts to His and Lys, respectively, on the F5 column. The corresponding chromatogram for a C_18_ column is shown in Figure S2.

Both columns, F5 and C_18_, gave symmetrical peaks, except for the peak corresponding to (−)-anti-BPDE-His-Pro, which exhibited tailing in both cases. The reason for this tailing behavior was not further investigated. A benefit with the F5 column, in contrast to C_18_, was the full separation of anti- from syn-adducts. All analyte peaks were resolved with F5, except for (+/−)-syn-BPDE-His-Pro, as shown in Figure 3. However, since measurement of the (+)-anti-BPDE adducts was considered as the most important, the obtained separation was judged to be acceptable for the present study. Due to the higher separation efficiency of the F5 column it was applied in the measurement of BPDE adducts in in vivo samples of both mice and human SA (cf. Section 3.4).

### 3.3. Method Evaluation

**Figure 4:**
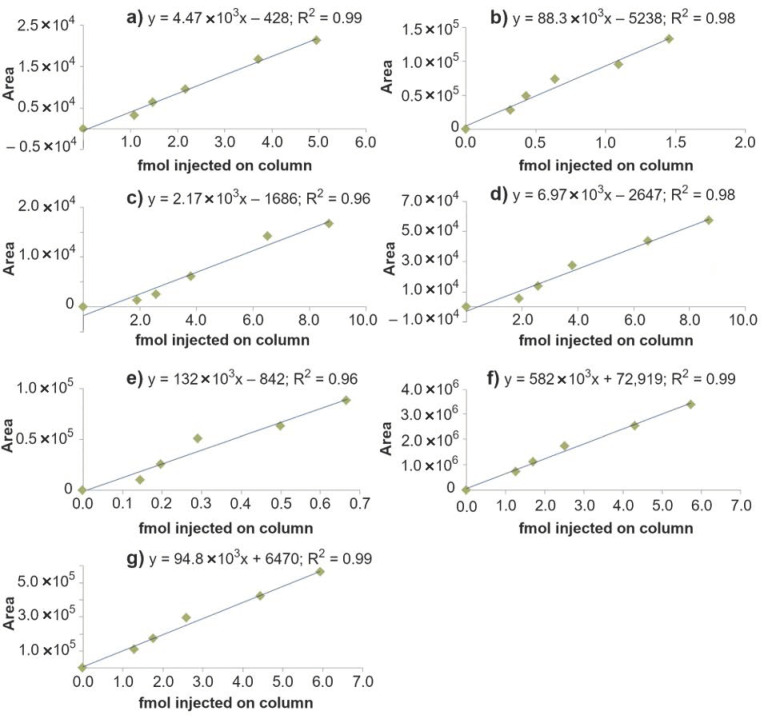
Linear regression curves of (**a**) (+)-anti-BPDE-Lys; (**b**) (−)-anti-BPDE-Lys; (**c**) (+/−)-syn BPDE-Lys (peak 1, retention time 9.0 min); (**d**) (+/−)-syn BPDE-Lys (peak 2, retention time 9, 9 min); (**e**) (+)-anti BPDE-His-Pro; (**f**) (−)-anti BPDE-His-Pro; and (**g**) (+/−)-syn BPDE-His-Pro. Different concentration levels were achieved by dilutions of in vitro alkylated human SA (see Section 2.3 and Section 2.4).

The calibration curves with obtained linearity for BPDE-His-Pro and BPDE-Lys isomers are shown in Figure 4 and the results summarized in Table 2. Ranges evaluated were from 0.1 to 10 fmol of the BPDE-adducts injected on column, corresponding to 5 to 500 fmol/mL. R^2^ was between 0.96 and 0.99 indicating a good linearity of the response. The mass accuracy of observed *m*/*z* values in human and mice samples was also evaluated. Deviations of less than 1 ppm of the observed *m*/*z* values from the corresponding theoretical values revealed that high mass accuracy data was obtained.

Method LODs are shown in Table 2 and were measured for the reference compounds generated from in vitro alkylated human SA. The obtained method LOD values for (+/−)-anti-BPDE-His-Pro, 0.004–0.008 fmol/mg SA, are about two orders of magnitude lower than those previously reported when using C_18_-HPLC/triple quadrupole MS, 1 fmol adduct/mg SA [19,23].

No carry-over in the analyses was detected as investigated from injection of solvent blanks between samples. The analysis of SA from non-exposed mouse (control) did not show the presence of His or Lys BPDE-adducts (Figure S4, Supplementary Material).

### 3.4. BPDE-Adducts In Vivo

#### 3.4.1. Measurements in Benzo[*a*]pyrene-Exposed Mice

Extracted ion chromatograms in Figure 3 from the F5-HPLC/ESI-Orbitrap MS/MS analyses show peaks corresponding to His-Pro (3b) and Lys (3e) adducts from the various BPDE isomers formed as metabolites in benzo[*a*]pyrene-exposed mice. Both (+/−)-syn and (+/−)-anti BPDE adducts could be detected. The use of HRMS, when combined with SPE enrichment, selective MS/MS ion transitions and the high separation efficiency of the F5 column, even with the tailing of (−)-anti-BPDE-His-Pro, considerably improved the separation and identification of target analytes from possible interferences in comparison to our earlier work [21,23]. On both F5 and C_18_ the elution was in agreement with that of the reference compounds. A chromatographic shift of about 0.1 min for the peak apex between in vivo adducts and the standards was indicated during the analysis. It should be noted, however, that the peaks are quite broad at the base (0.3–0.8 min approximately), and the bases are almost overlapping between standards and samples. Further, the Orbitrap data support *m*/*z* matches between standards and samples and strengthen the identification. To further gain confidence in the identity of the peaks, isotope-substituted internal standards with matching retention times and a similar fragmentation to the analytes would be optimal.

No internal standard was used in this study as the major aim was to resolve the adduct isomers and improve their detection limits, thus the measured adduct levels (quantified in comparison with calibration curves) are to be regarded as semi-quantitative. In our earlier development work [21], SA in vitro alkylated with a diol epoxide (DE) of another PAH compound was used as an internal standard for the quantification in the analysis of BPDE-His adducts in benzo[*a*]pyrene-exposed mice. Although such internal standard, consisting of PAH-DE alkylated SA, is not absolutely optimal for the evaluation of recovery and analytical accuracy, it has the advantage of reflecting the yield of enzymatic hydrolysis. This type of internal standard could be applied for further work, if generated from a PAH-DE or another bulky electrophile that is not present as background exposure.

Table 3 lists the estimated levels of (+)-anti- and (−)-anti-BPDE adducts in SA from the exposed mice (100 mg benzo[*a*]pyrene/kg of body weight) at day 3 after treatment. These data show relatively large variations between the mice but consistently indicate a higher adduct level from (+)-anti-BPDE both to His and Lys than of (−)-anti-BPDE. Samples were taken at different time points, at 1, 3, 7, 14 and 28 days after exposure. Figure S5 shows the estimated levels of His and Lys adducts at the different days. A maximum can be observed at day 3, which is in agreement with what was indicated in our earlier work [21]. This reflects a rather fast metabolism of benzo[*a*]pyrene to BPDE and subsequent formation of protein adducts as well as an elimination of studied adducts that follows the half-life of mouse SA (i.e., about 1.9 days [33]). It is worth noting that the adduct levels in the samples at day 28 after exposure are below LOD, which was expected due to the short half-life of SA in mice. The estimated range of the (+)-anti-BPDE-His adduct levels was 0.1–0.3 fmol/mg, while the (−)-anti-BPDE-His adduct levels were about half (Table 3). The levels of (+/−)-syn adducts were estimated to be at least 10 times higher (cf. Figure 3 and Figure 4). 

To be able to compare our present results with earlier results [21,23] obtained during development of this method, we made a rough estimate of the total BPDE-His adduct level in the present study by summing (+/−)-anti- and (+/−)-syn-BPDE-His and adjusting for ca. 50% SA content in the samples (see Section 2.2). In two earlier studies, analyses of the total BPDE-His adduct levels were preliminary quantified in mice treated with the same dose of benzo[*a*]pyrene (100 mg/kg body weight; mice from the same experiment or mice of different strain from another experiment, sampling 3 or 2 days after exposure, respectively) [21,23]. The approximately estimated total BPDE-His adduct levels in these two studies are higher, but less than three times higher than in the present study. In both these earlier studies, we used triple quadrupole MS instrumentation and insufficient chromatographic separation of the BPDE-His adduct isomers using C_18_ as HPLC stationary phase, even though in the latest previous study [23], we used conditions of somewhat resolved (+)-anti-BPDE-His adducts. In that study, we also preliminary estimated the (+)-anti-BPDE-His adducts for the first time, to a level about 10 times higher than obtained in the present study with improved quantification. We judge that the previous figures on adduct levels can be prone to uncertainty, particularly the level of the (+)-anti-BPDE-His adduct, corresponding to a minor peak. The lower levels obtained in the present study indicate the importance of improved mass resolution and accuracy as in the present MS analysis as well as the higher chromatographic selectivity of the F5 column for resolving peaks and quantification of adduct levels. Selection of internal standards is another important issue discussed above.

#### 3.4.2. Detection of Adducts in Human Serum Albumin

Adducts from BPDE were detected in only two of the twelve human SA samples, and that from (+)-anti-BPDE was detected in only one. The chromatograms from the latter sample indicating the peaks of BPDE-His-Pro and BPDE-Lys are shown in Figure 3c,f, respectively. A major peak corresponding to (−)-anti-BPDE-His-Pro along with a minor peak for (+)-anti-BPDE-His-Pro can be observed in Figure 3c. Of these adducts, only the level of (−)-anti-BPDE-His-Pro could be quantified and was estimated to be 0.11 fmol/mg of SA. Only one Lys adduct, (−)-anti-BPDE-Lys, could be detected as shown in Figure 3f, and its level was approximately 0.13 fmol/mg. The lack of a peak corresponding to (+)-anti-BPDE-Lys might be explained by a 20-fold higher method LOD as compared to the (-)-anti isomer (cf. Table 2). In the other adduct-positive human sample, only (−)-anti-BPDE-His-Pro was observed but could not be quantified (Figure S6 in Supplementary Material).

It is notable that an adduct formed from the most carcinogenic BPDE isomer, (+)-anti-BPDE [22], could be detected in one of the human SA samples with the presented method. Adducts from (+/−)-syn BPDE were not detected in any of the two positive human samples, unlike the samples from benzo[*a*]pyrene-exposed mice (Figure 3). The detection of (−)-anti-BPDE-His-Pro in the two human samples is in agreement with the only earlier observation (by laser-induced fluorescence) of adducts to His in humans. That study showed the occurrence of (−)-anti-BPDE as the only isomer, with a median adduct level of 0.16 fmol/mg (from 63 samples from the general population) [15]. Individual differences in the metabolism of benzo[*a*]pyrene in humans agree with the results reported in other publications, in which DNA adduct levels and polymorphism in metabolizing genes have been studied after experimental exposure to [^14^C]-benzo[*a*]pyrene [34] or after therapeutic treatment with coal tar involving benzo[*a*]pyrene exposure [35].

Considering the much lower exposures to benzo[*a*]pyrene in human than in the treated mice, the indicated ranges of adduct levels in humans can be regarded as relatively high. As a comparison to the mice exposure in the present study, the mean intake of benzo[*a*]pyrene from food in Sweden has recently been estimated to be 50 ng per person per day [3]. On the other hand, the comparison between species should take into account that the half-life of SA is much longer for humans, about 20 days, than for mice (a few days), and thus stable adducts are accumulated to a steady-state during chronic exposure in humans [12]. In this regard, it is also of interest to clearly differentiate between different approaches to assess exposure to benzo[*a*]pyrene in vivo. Unlike the method proposed here that measures adducts bound to the reactive nucleophilic sites in the protein, i.e., His146 or Lys195, it has been a strategy to measure benzo[*a*]pyrene tetraols, either hydrolyzed from protein or DNA or as urine metabolites [13,16,36]. Recently, an approach using chiral HPLC, followed by GC/electron-capture negative ionization–tandem MS, has been applied to quantify enantiomeric tetraols in human urine. The compounds could be detected in samples from all individuals due to the high sensitivity of the MS method [36]. However, in this method the measured analytes represent the excreted metabolites, giving no estimate of the in vivo dose of the specific diol epoxides. During the course of the present work, one research group has been able to measure BPDE adducts to deoxyguanosine (BPDE-dG) in human umbilical cord using triple quadrupole MS [37]. The DNA adducts from (+/−)-anti-BPDE were detected in all samples (n = 84), which means a large step forward for a methodology for specific measurement of DNA adducts from BPDE in humans. The BPDE-dG adduct levels were found to be several orders of magnitude higher [37] (in mol per g) than the human BPDE-His-Pro levels estimated in the present work. This is in accordance with our earlier preliminary comparison of the levels of these two types of adducts, i.e., ratio of the DNA-to-SA adducts, in the studied benzo[*a*]pyrene exposed mice [21,23].

## 4. Summary and Future Perspectives

The present study has demonstrated an HPLC/HRMS/MS methodology to detect and identify BPDE-His adducts at the sub-fmol/mg SA level in both benzo[*a*]pyrene-exposed mice and in humans. MS has the benefit of inherent high specificity in the identification compared to ELISA or laser-induced fluorescence. Furthermore, the results show the ability of high-resolution and high-mass accuracy MS instrumentation, such as Orbitrap, to reach lower LODs. Another improvement is the use of a more selective chromatographic separation, in this case by employing an F5 column to separate the adduct isomers and to reduce the number of interfering compounds in the MS analyses. In the present study, chromatographic separation between adducts from BPDE enantiomers was obtained without the need for a chiral column.

It was shown to be possible to detect SA adducts of BPDE in human samples, although the His adduct from the most carcinogenic (+)-anti-BPDE enantiomer was detected in only one out of the twelve individual samples. BPDE-Lys adducts (Lys195 as earlier shown [17]) were detected in the samples from exposed mice and in one human. BPDE-Lys adducts are previously not specifically quantified in in vivo samples and should be further evaluated for quantification of BPDE isomers. 

The method is semi-quantitative and warrants further validation, particularly with regard to quantification of absolute levels. In this work the focus was on separation and detection of the BPDE-adducts. For the approach of using SA to measure exposure to reactive metabolites of PAHs, further work is required to validate the quantitative aspects particularly at the low levels expected in the general population. Important for future studies is the availability of an internal standard particularly for the (+)-anti-BPDE-His adduct. Isotope-substituted internal standards are to be preferred, although they are costly to synthesize. However, it should be considered that for BPDE several stereoisomeric adducts are formed, that adducts both to His and Lys would be of value to quantify, and further that exposure to several PAH might be possible to measure. This would require a pragmatic solution of a common internal standard for several adducts, which could adjust for digestion efficiency.

One goal has been to use a relatively simple work-up for the samples which is applicable for analysis of a broad range of bulky adducts, particularly from PAHs, which means, e.g., that specific antibodies^17^ have not been the choice for enrichment of adducts. For application to human samples in large numbers, it would be an advantage if the precipitation with ammonium sulphate could be excluded, and methods for isolation [38] adapted for high throughput are evaluated. Moreover, that the analyte is an adduct to His-Pro might be an advantage compared to an adduct to Lys, as the peptide His-Pro would be a more specific marker for adduct formation with SA. In addition, BPDE adducts to His in Hb could be further explored. Adduct formation in vitro and in vivo has been studied previously [18,39], which indicated that His adduct levels in Hb are ca. 10 times lower than in SA in exposed mice (exposure to radiolabeled benzo[*a*]pyrene) [39].

The presented methodology, with some further improvements as suggested above, opens the way for applications to characterize benzo[*a*]pyrene-exposure in humans and also for further comparison with exposed mice for species extrapolation, with regard to in vivo doses of genotoxic metabolites and risk from exposure to benzo[*a*]pyrene. However, the levels of BPDE adducts to His estimated in this study in one human sample in vivo are very low, in the order of 1 mole of adduct in 10^8^ moles of SA, and thus, it might be challenging to quantify levels of these adducts within a population without high exposure levels, such as the general population exposed via common diet and inhalation of urban air pollution. On the other hand, it might still be possible to further improve the detection limits, such as by using a more selective SPE stationary phase for an efficient enrichment of BPDE adducts. Nevertheless, we have here shown that using HRMS and a chromatographic column like F5, it should be possible to determine different stereoisomeric BPDE-adducts in humans exposed to benzo[*a*]pyrene.

## Figures and Tables

**Table 1:** Identification of BPDE adducts in in vitro alkylated SA based on elemental composition of the [M + H]^+^ ions according to accurate *m*/*z* measurements.

BPDE Isomer	Adducted to	Elemental Composition	Theoretical *m*/*z*	Measured *m*/*z*	Δ ppm	DBE ^a^
(+)-anti	His-Pro	C_31_H_31_O_6_N_4_	555.2238	555.2240	0.27	18.5
(−)-anti		C_31_H_31_O_6_N_4_	555.2238	555.2238	−0.02	18.5
(+)-anti	Lys	C_26_H_29_O_5_N_2_	449.2071	449.2067	−0.80	13.5
(−)-anti		C_26_H_29_O_5_N_2_	449.2071	449.2069	−0.47	13.5

^a^ Double-bond equivalents (DBE) = C + 1 + (N/2) − (H/2), where C, N and H are the number of carbon-, nitrogen- and hydrogen atoms, respectively. Half-integer values correspond to even electron ions (in this case protonated molecules).

**Table 2:** Method evaluation parameters, calculated for the different BPDE adducts to His (A) and Lys (B) using in vitro alkylated human SA in solution.

**A. Adducts to His**.						
**BPDE-His-Pro, Precursor Ion *m*/*z* 555.2, [M + H]^+^**						
**BPDE Isomer**	**Retention Time (min)**	**Linear Range** **(fmol Adduct Injected)**	**R^2^** **(n = 5)**	**LOD ^a^** **(fmol/mg SA)**	***m*/*z* of Product ion**	**Δ ppm**
(+)-anti-	9.2	0.1–0.7	0.96	0.004	257.0958	−1.17
(−)-anti-	9.8	1.3–5.7	0.99	0.008	257.0960	−0.39
(+/−)-syn-	9.5	1.3–5.9	0.99	0.008	257.0959	−0.78
**B. Adducts to Lys**.						
**BPDE-Lys, Precursor Ion *m*/*z* 449.2, [M + H]^+^**						
**BPDE Isomer**	**Retention Time (min)**	**Linear Range** **(fmol Adduct Injected)**	**R^2^** **(n = 5)**	**LOD ^a^** **(fmol/mg SA)**	***m*/*z* of** **Product ion**	**Δ ppm**
(+)-anti-	8.5	1.1−5.0	0.99	0.2	257.0959	−0.78
(−)-anti-	8.7	0.3−1.5	0.98	0.01	257.0959	−0.78
(+/−)-syn- (isomer 1) ^b^	9.0	na	na	na	257.0960	−0.39
(+/−)-syn- (isomer 2) ^b^	9.9	na	na	na	257.0959	−0.78

^a^ Method LOD obtained from instrumental LOD at S/N = 3, estimated from the standard deviation from five injections of diluted working solutions (blank was without signal). ^b^ Lys adduct ratios for (+) to (−)-syn-BPDE were not known and therefore quantification was not available (na).

**Table 3:** Levels of adducts from (+)-anti- and (−)-anti-BPDE in mice euthanized 3 days after treatment with benzo[*a*]pyrene (100 mg/kg of body weight). (Two individual mice were analyzed.)

	BPDE-His-Pro	BPDE-Lys
BPDE isomer	Adduct levels (fmol/mg SA) ^a^ N = 2	Adduct levels (fmol/mg SA) ^a^ N = 2
(+)-anti-	0.11/0.30	0.71/2.8
(−)-anti-	0.095/0.11	0.083/< LOD

^a^ Levels not adjusted for ca 50% content of salt in SA samples.

## Data Availability

The data presented in this study are available in the main article and supplementary material.

## Supplementary Materials

The following supporting information can be downloaded at: https://www.mdpi.com/article/10.3390/toxics10010027/s1, Figure S1: MS/MS spectra showing fragmentation pattern of standard compounds of (**a**) (+/-)-BPDE-His-Pro and (b) (+/-)-BPDE-Lys obtained from *in vitro* alkylated human SA; Figure S2: Extracted ion chromatograms using the common fragment ion at *m*/*z* 257.0961 showing separation of the studied adducts from the standard of *in vitro* alkylated SA. Separation was done on a C_18_-HPLC analytical column and employing Orbitrap tandem HRMS (PRM mode). a) Lys adducts and b) His adducts, with [M+H]^+^ monitored at *m*/*z* 449.2 and 555.2, respectively; Figure S3: Extracted ion chromatograms, showing (+/-)-anti-BPDE-adduct references from the standard of *in vitro* alkylated SA, employing a C_18_ column coupled to triple-quadrupole MS/MS (MRM mode). Lys adducts (a) and His-adducts (b) are observed (with a poor signal compared to that observed with Orbitrap MS, as shown in Figure S2); Figure S4: Extracted ion chromatogram of SA from control mouse (non-alkylated, 10 mg SA); Figure S5: Levels of adducts to His (a) and Lys (b) from (+)-anti-BPDE in mice euthanized at different days after exposure to benzo[a]pyrene (100 mg/kg of body weight); Figure S6: Extracted ion chromatogram showing (-)-anti-BPDE-His-Pro in a second human SA (10 mg) sample. Separation was performed on an F5 HPLC column and using Orbitrap tandem HRMS (PRM mode).
