# Supplementary material for: Detection of Benzo[a]pyrene Diol Epoxide Adducts to Histidine and Lysine in Serum Albumin In Vivo by High-Resolution-Tandem Mass Spectrometry
Source: Toxics. 2022 Jan 8;10(1):27. doi: 10.3390/toxics10010027 (PMC8778559; doi:10.3390/toxics10010027)
Supplement: Supplementary file 1 [file toxics-10-00027-s001.zip › toxics-1501042-supplementary.pdf]

Supplementary Materials: Detection of benzo[a]pyrene diol epoxide adducts to histidine and lysine in serum albumin in vivo by high-resolution-tandem mass spectrometry

Javier Zurita, Hitesh V. Motwani, Leopold L. Ilag, Vassilis L. Souliotis, Soterios A. Kyrtopoulos, Ulrika Nilsson and Margareta Törnqvist

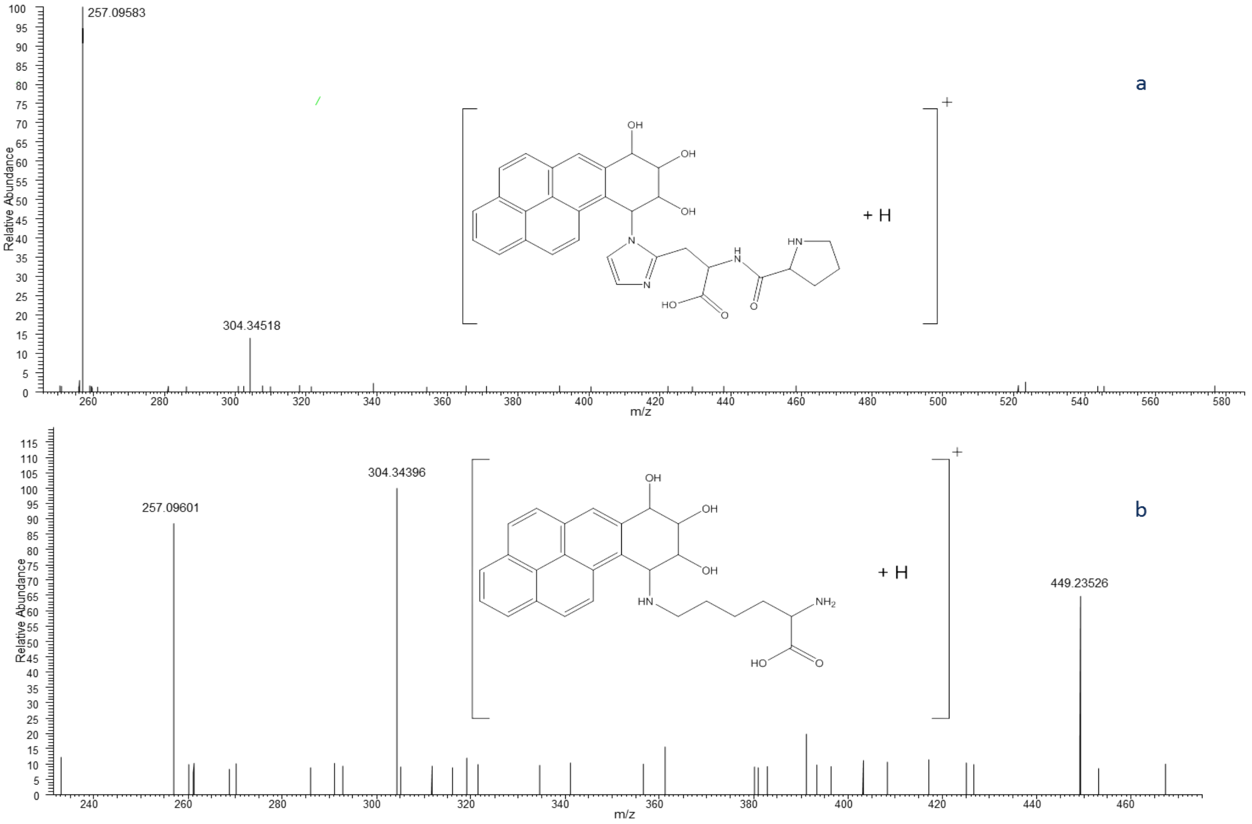

Figure S1. MS/MS spectra showing fragmentation pattern of standard compounds of (**a**) (+/-)-BPDE-His-Pro and (**b**) (+/-)-BPDE-Lys obtained from *in vitro* alkylated human SA.

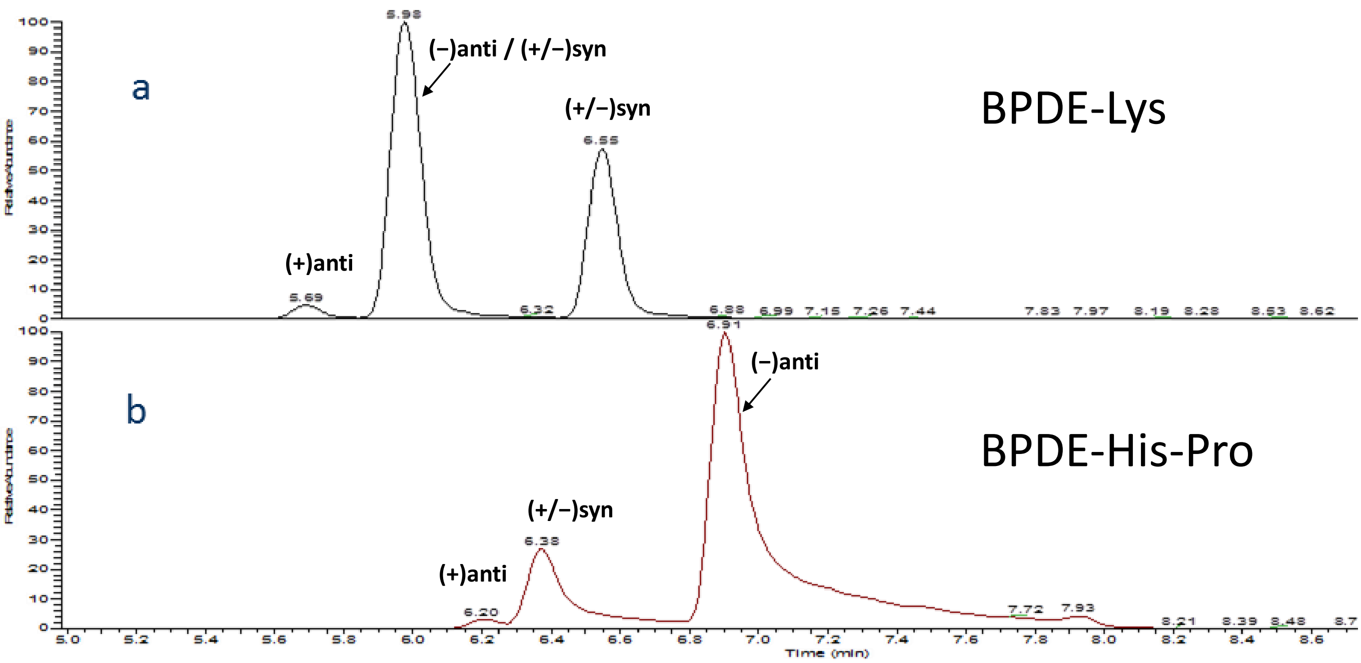

Figure S2. Extracted ion chromatograms using the common fragment ion at m/z 257.0961 showing separation of the studied adducts from the standard of *in vitro* alkylated SA. Separation was done on a C_{18}-HPLC analytical column and employing Orbitrap tandem HRMS (PRM mode). **a**) Lys adducts and **b**) His adducts, with $[M + H]^+$ monitored at m/z 449.2 and 555.2, respectively.

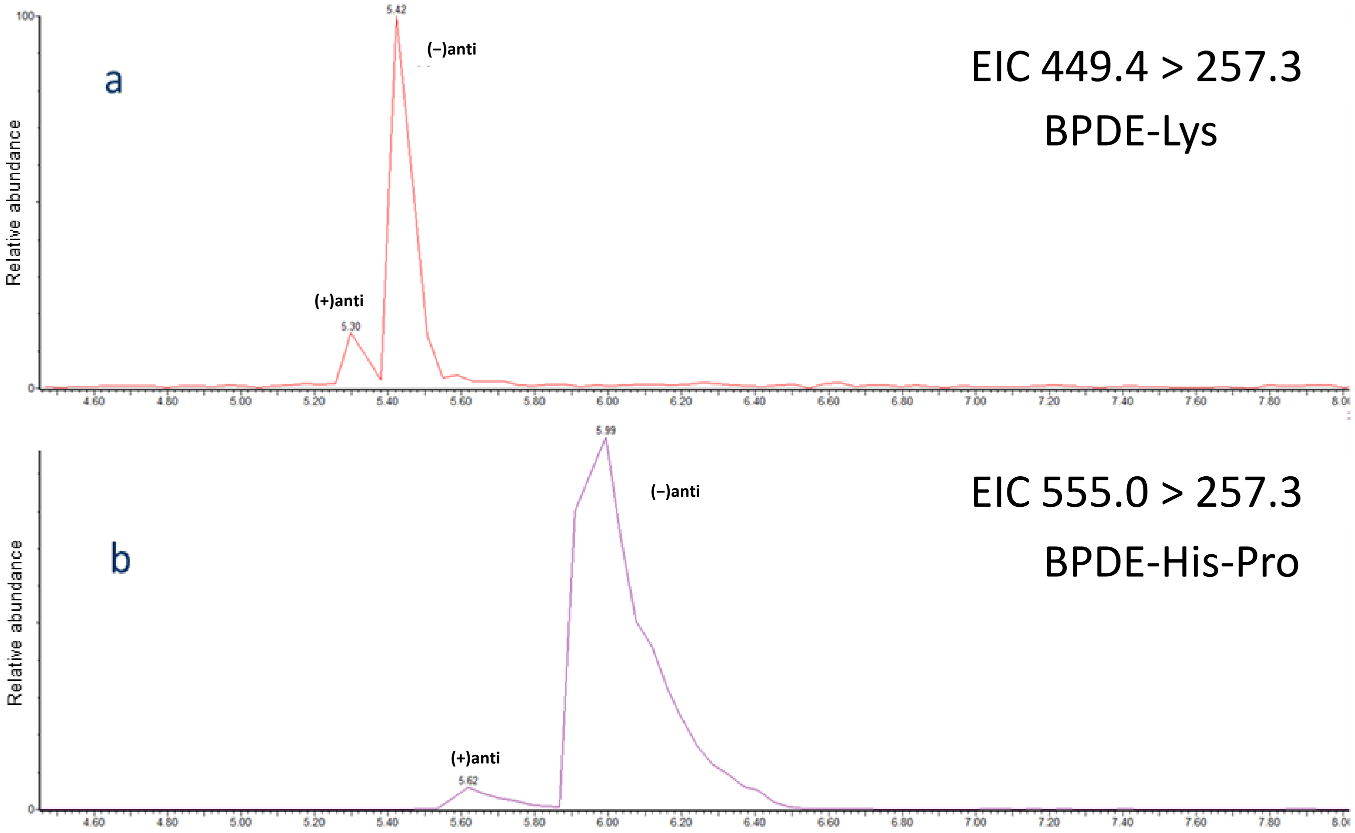

Figure S3. Extracted ion chromatograms, showing (+/−)-anti-BPDE-adduct references from the standard of *in vitro* alkylated SA, employing a C_{18} column coupled to triple-quadrupole MS/MS (MRM mode). Lys adducts (**a**) and His adducts (**b**) are observed (with a poor signal compared to that observed with Orbitrap MS, as shown in Figure S2).

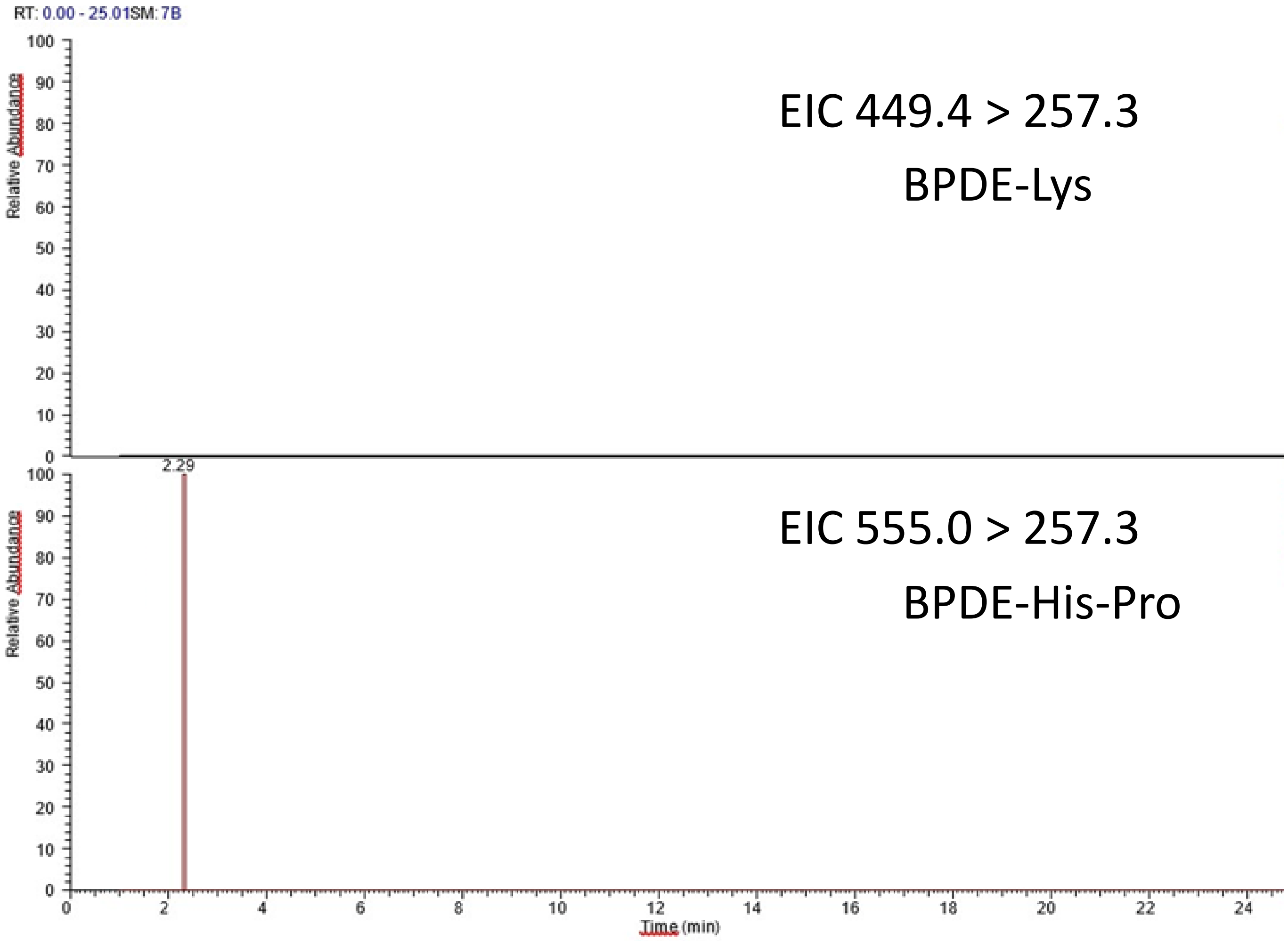

Figure S4. Extracted ion chromatogram of SA from control mouse (non-alkylated, 10 mg SA).

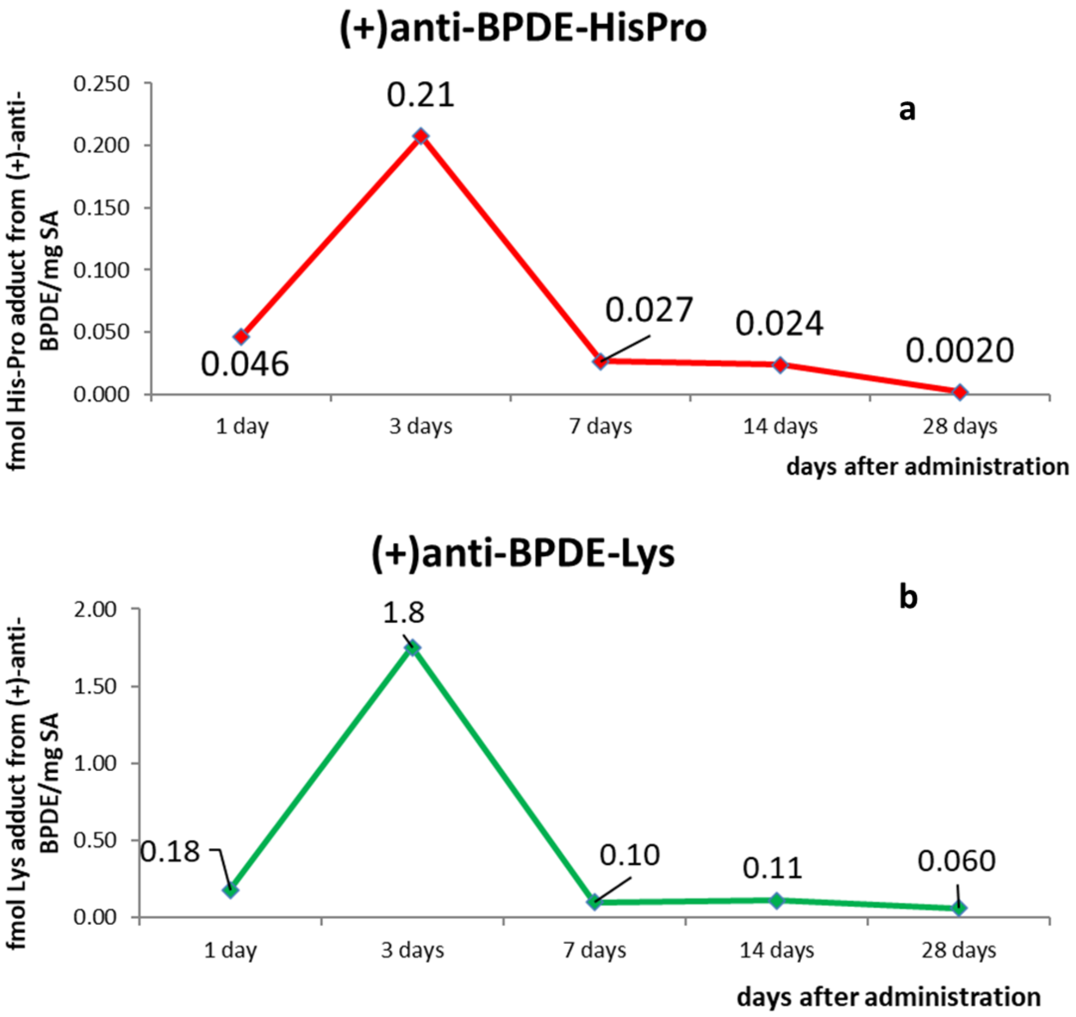

Figure S5. Levels of adducts to His (**a**) and Lys (**b**) from (+)-anti-BPDE in mice euthanized at different days after exposure to benzo[a]pyrene (100 mg/kg of body weight). Mean values in two exposed mice from each day are shown (half LOD used when level below LOD).

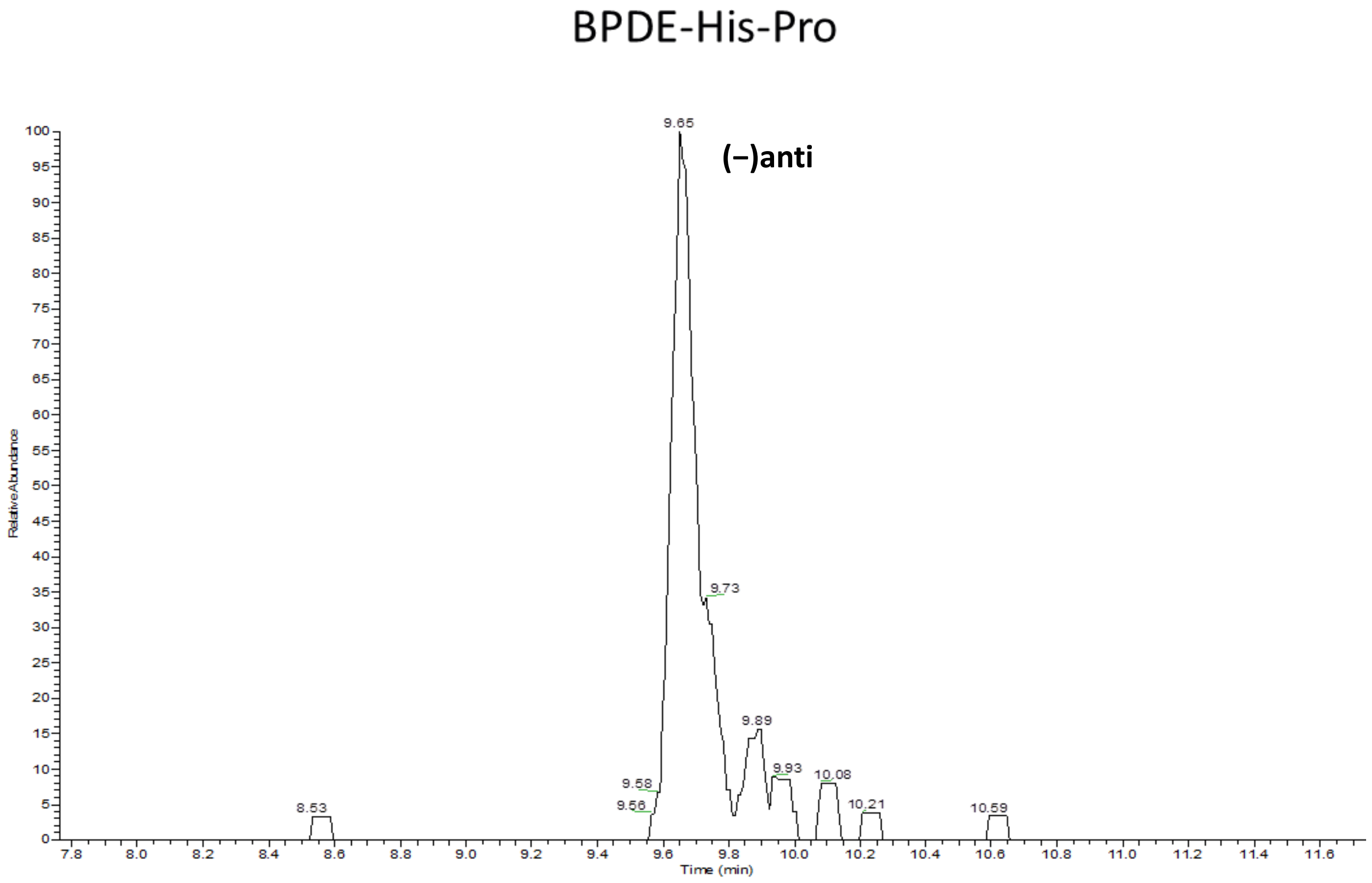

Figure S6. Extracted ion chromatogram showing (−)-anti-BPDE-His-Pro in a second human SA (10 mg) sample. Separation was performed on an F5 HPLC column and using Orbitrap tandem HRMS (PRM mode).
